# Effects of IL-38 on Macrophages and Myocardial Ischemic Injury

**DOI:** 10.3389/fimmu.2022.894002

**Published:** 2022-05-13

**Authors:** Zhiyang Li, Yan Ding, Yudong Peng, Jian Yu, Chengliang Pan, Yifan Cai, Qian Dong, Yucheng Zhong, Ruirui Zhu, Kunwu Yu, Qiutang Zeng

**Affiliations:** Department of Cardiology, Union Hospital, Tongji Medical College, Huazhong University of Science and Technology, Wuhan, China

**Keywords:** interleukin-38, macrophages, myocardial infarction, myocardial ischemia–reperfusion injury, ventricular remodeling, inflammation

## Abstract

Macrophages play an important role in clearing necrotic myocardial tissues, myocardial ischemia–reperfusion injury, and ventricular remodeling after myocardial infarction. M1 macrophages not only participate in the inflammatory response in myocardial tissues after infarction, which causes heart damage, but also exert a protective effect on the heart during ischemia. In contrast, M2 macrophages exhibit anti-inflammatory and tissue repair properties by inducing the production of high levels of anti-inflammatory cytokines and fibro-progenitor cells. Interleukin (IL)-38, a new member of the IL-1 family, has been reported to modulate the IL-36 signaling pathway by playing a role similar to that of the IL-36 receptor antagonist, which also affects the production and secretion of macrophage-related inflammatory factors that play an anti-inflammatory role. IL-38 can relieve myocardial ischemia–reperfusion injury by promoting the differentiation of M1 macrophages into M2 macrophages, inhibit the activation of NOD-like receptor thermal protein domain-associated protein 3 (NLRP3) inflammasome, and increase the secretion of anti-inflammatory cytokines, such as IL-10 and transforming growth factor-β. The intact recombinant IL-38 can also bind to interleukin 1 receptor accessory protein-like 1 (IL-1RAPL1) to activate the c-jun N-terminal kinase/activator protein 1 (JNK/AP1) pathway and increase the production of IL-6. In addition, IL-38 regulates dendritic cell-induced cardiac regulatory T cells, thereby regulating macrophage polarization and improving ventricular remodeling after myocardial infarction. Accordingly, we speculated that IL-38 and macrophage regulation may be therapeutic targets for ameliorating myocardial ischemic injury and ventricular remodeling after myocardial infarction. However, the specific mechanism of the IL-38 action warrants further investigation.

## Introduction

Myocardial infarction (MI), an ischemic injury of the myocardium following the obstruction of coronary artery flow, is one of the leading causes of death worldwide and is closely related to inflammation. After MI, various inflammatory mediators released by the ischemic myocardium are involved in tissue repair and related adaptive responses (1–4). The development of necrotic myocardium after MI can be categorized into 3 overlapping stages: the inflammatory stage, the proliferative stage, and the mature stage. During the inflammatory phase, the activated cascades of cytokines, including the Nuclear factor kappa B (NF-kB) system and the complement system, enable the recruitment of neutrophils and macrophages to the infarcted area and clear the necrotic cells. During the proliferation phase, activated macrophages release cytokines and growth factors, such as transforming growth factor (TGF)-β and interleukin (IL)-10, resulting in the formation of highly vascularized granulation tissues. Meanwhile, the expression of pro-inflammatory mediators is inhibited, and fibroblasts and endothelial cells begin to proliferate. The activated myofibroblasts produce extracellular matrix proteins that form extensive microvascular networks. After entering the mature stage, fibroblasts and vascular cells undergo apoptosis, leading to collagen scarring, namely, ventricular remodeling (5, 6). Furthermore, the continuous excessive inflammatory response increases collagen deposition and matrix degradation, thereby aggravating ventricular remodeling and seriously affecting cardiac functions later in life. The inflammatory cascade induced by ischemia–myocardial reperfusion therapy after MI can also damage the myocardial tissue to a certain extent (7). Research evidence suggests that effective inhibition of an excessive inflammatory response after MI can reduce myocardial ischemia injury and improve cardiac function (5, 8). As a newly discovered inflammatory factor, IL-38 has been extensively studied for its anti-inflammatory effects. In our previous study, plasma IL-38 levels were significantly increased in a time-dependent manner in patients with ST-segment elevation MI (9). Animal experiments showed that IL-38 can exhibit an anti-inflammatory effect by regulating macrophage function (10, 11). Therefore, the regulation of the effect of IL-38 on macrophages in myocardial ischemic injury might be considered a new direction for the treatment of MI and the improvement of its prognosis.

## Interleukin-38 and Macrophages

**Figure 1:**
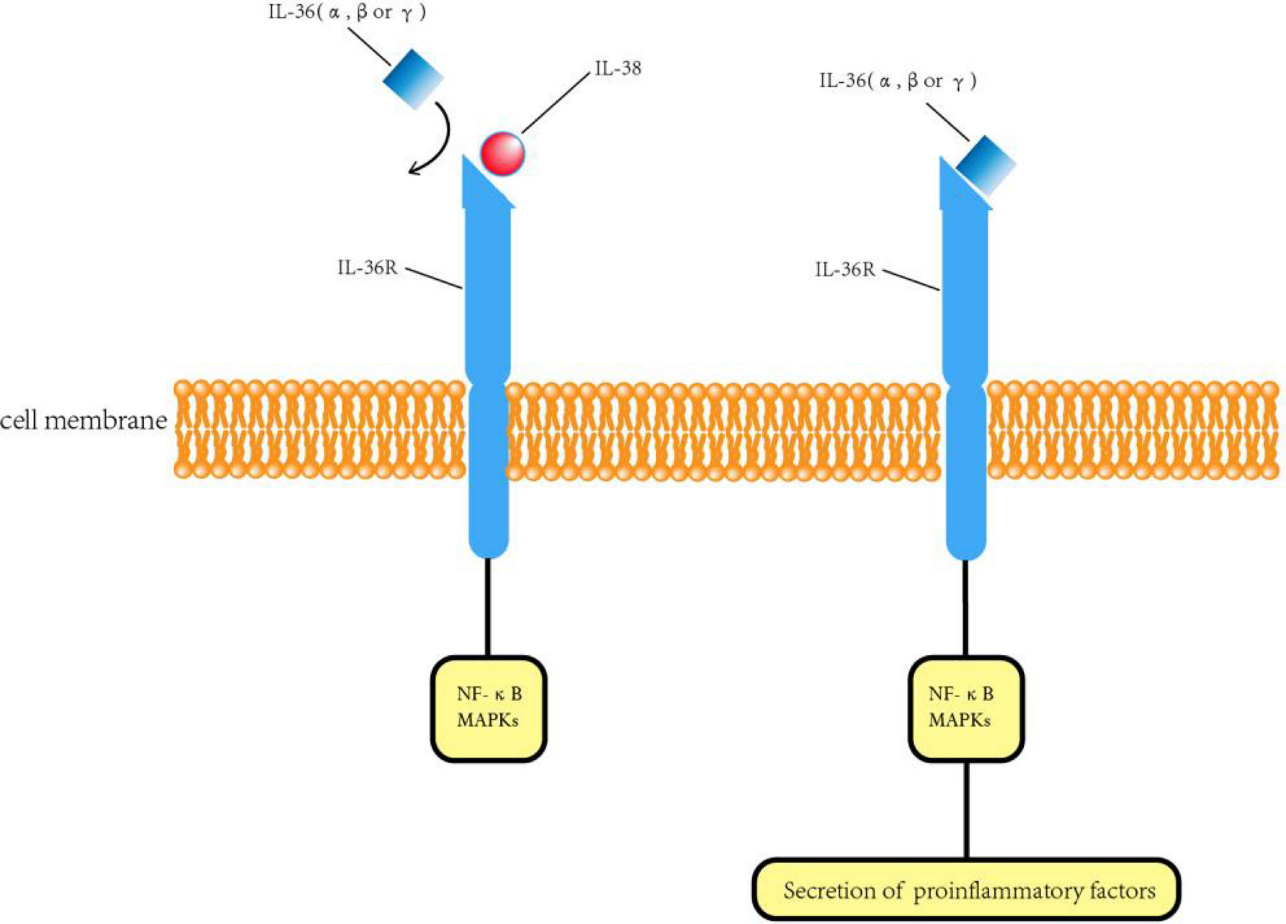
Regulation of IL-38 on the IL-36 signaling pathway. IL-38, interleukin-38; IL-36, interleukin-36; IL-36R, interleukin-36 receptor; MAPKs, Mitogen-activated protein kinases; NF-kB, Nuclear factor kappa B.

IL-38, the 10th member of the IL-1 family, is widely expressed in human organs and tissues such as the placenta, spleen, thymus, and tonsil. It is most abundantly involved in B-cell proliferation in the skin and tonsil, whereas it is present in low levels in the heart and other non-immune organs (12). Close to the genes encoding IL-1 receptor antagonist (IL-1Ra) and IL-36 receptor antagonist (IL-36Ra), the gene encoding IL-38 is located on human chromosome 2Q13-14.1 (13). This cytokine is reported to have 37% (13) and 41% homology with IL-1Ra and 43% homology with IL-36Ra (14, 15). Van de Veerdonk et al. (16) suggested that the biological function of IL-38 is similar to that of IL-36Ra. In other words, it competitively binds to the IL-36 receptor (IL-36R) and inhibits the binding of IL-36 cytokines [IL-1F6 (IL-36α), IL-1F8 (IL-36 β), IL-1F9 (IL-36 γ)] with IL-36R and thereby affects the subsequent activation of NF-κB and Mitogen-activated protein kinase (MAPK), thus exhibiting certain anti-inflammatory effects (16, 17) (**Figure 1**). Previous studies on the association between IL-38 allele polymorphism and diseases have reported a relationship between IL-38 in compulsive arthritis and psoriatic arthritis (18, 19). Recent studies have reported IL-38 in human coronary atherosclerotic plaques (20). Moreover, polymorphisms in IL-38 are associated with coronary artery disease (21) and C-reactive protein (CRP) concentrations in humans (22). These results suggested that IL-38 may play an important role in coronary artery disease.

Macrophages, which are the main cells in inflammation, are functionally divided into classically activated macrophages (M1) associated with inflammatory responses and alternatively activated macrophages (M2) associated with injury repair and regeneration (23). At the onset of the inflammatory phase of MI, M1 macrophages, activated by tumor necrosis factor (TNF)-α, interferon (IFN)-γ, and lipopolysaccharide (LPS), are the major subtypes in the removal of dead cells and extracellular matrix debris (24, 25). In the proliferative stage of MI, M2 macrophages promote the repair and regeneration of damaged heart tissue (26). Notably, recent studies have reported that IL-38 can affect the production and secretion of inflammatory factors related to macrophages, thus playing a relevant role in systemic lupus erythematosus (27), arthritis (28), mandatory spondylitis (29), and cardiovascular diseases (9). Therefore, IL-38 is expected to be a new therapeutic target for ischemic heart injury *via* the regulation of macrophages.

## Role of Interleukin-38 in Myocardial Infarction

MI is one of the cardiovascular events associated with high mortality worldwide. After MI, inflammatory mediators are released from the myocardium as a response to injury, post-injury repair, and adaptation (1–4). Our previous study reported that plasma IL-38 levels in patients with ST-segment elevation MI are positively correlated with CRP, cardiac troponin I (cTNI), and N-terminal pro-brain natriuretic peptide (NT-proBNP) but weakly negatively correlated with left ventricular ejection fraction (9). cTNI has been widely studied as a diagnostic and prognostic marker of the acute coronary syndrome (ACS), and a previous study has reported that patients with ACS with anti-cTNI autoantibodies will eventually trigger severe autoimmune inflammation if mistaken by the immune system for antigens rather than self-protein (30). Thus, cTNI can not only represent the degree of cardiac injury but also predict autoimmune inflammation after Acute myocardial infarction (AMI). Because of its positive correlation with cTNI levels, IL-38 levels may be an indicator of myocardial injury or autoimmunity. Increased serum CRP levels are a nonspecific but sensitive marker of an acute inflammatory response, and high levels of CRP after acute MI can predict infarct expansion, cardiac rupture, and mortality (31, 32). Altogether, plasma IL-38 levels may be a new indicator to determine whether percutaneous coronary intervention is successful or not and can reflect the degree of the anti-inflammatory response *in vivo*. In addition, Zare Rafie et al. (33) showed that increased serum IL-38 levels were observed 24 h after tissue plasminogen activator treatment in patients with ischemic stroke, and a significant correlation was observed between changes in IL-38 concentration and patients’ prognosis at 3 months. The mechanisms underlying ischemic stroke and MI are blood flow obstruction and subsequent tissue inflammation. These results suggested that changes in serum IL-38 levels may be a new early predictor of the prognosis of ischemic disease.

## Interleukin-38 Modulates Macrophage Functions After Myocardial Infarction

**Figure 2:**
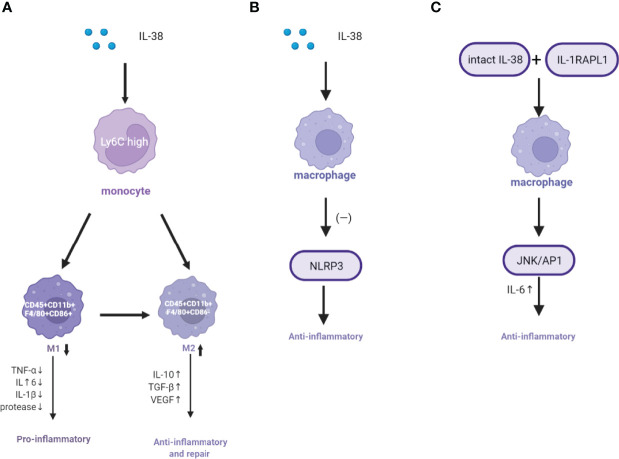
Mechanism of IL-38 in regulating the macrophage function. **(A)** IL-38 regulates the phenotypic transformation of macrophages. **(B)** IL-38 inhibits NLRP3 activation. **(C)** IL-38 binds IL-1RAPL1 to regulate the macrophage function. IL-38, interleukin-38; TNF-α, tumor necrosis factor-α; VEGF, vascular endothelial growth factor; TGF-β, transforming growth factor-β; NLRP3, NOD-like receptor thermal protein domain-associated protein 3; IL1RAPL1, interleukin-1 receptor accessory protein-like 1 (–); inhibition.

After MI, morphological changes occur in the infarcted and non-infarcted areas of the ventricle, which affect the pumping function of the heart and eventually lead to heart failure (34). In this process, inflammation plays an important role in the changes in myocardial tissue structure after MI (35, 36). As key cells involved in inflammation, M1 macrophages mainly secrete TNF-α, IL-1β, and IL-6 to promote inflammation, whereas M2 macrophages mainly secrete IL-10 and TGF-β, which are related to inflammation regression and tissue repair and remodeling (37, 38). Among the inflammatory markers, IL-6 is associated with adverse clinical outcomes as reported in previous clinical trials, whereas increased levels of the anti-inflammatory cytokine IL-10 are associated with more favorable outcomes (39, 40). When MI occurs, the ischemic myocardial tissue rapidly recruits inflammatory monocytes with a high expression of Ly6C and reaches a peak 3 days after MI. The recruited monocytes differentiate into inflammatory macrophages in response to chemokines and inflammatory cytokines (41, 42). Pro-inflammatory M1 macrophages secrete several inflammatory factors such as TNF-α, IL-1 β, and proteases that help clarify dead cells and debris from the infarcted areas. However, the long-term inflammatory response caused by these compounds can lead to extensive damage to the infarcted myocardium, thus affecting subsequent ventricular remodeling (43–45). From 4 to 14 days after MI, macrophages gradually change from inflammatory (M1) to anti-inflammatory or repair (M2). M2 macrophages establish an anti-inflammatory environment by downregulating inflammatory cytokines and upregulating anti-inflammatory cytokines such as IL-10, TGF-β, and vascular endothelial growth factor, and the inflammatory response is gradually replaced by angiogenesis and myofibroblast differentiation (46). Previous studies have reported that IL-38 plays an anti-inflammatory role by binding to the IL-36R and neutralizing the IL-36 inflammatory factor signaling pathway (16). *In vitro* culture of THP-1 macrophage cell line showed that IL-38 overexpression can effectively reduce the expression of IL-6, TNF-α, IL-23, and IL-10 induced by LPS but did not alter the expression of IL-1β (28). Ge et al. (11) observed that IL-38 promoted the differentiation of macrophages into anti-inflammatory M2 macrophages but prevented the differentiation into pro-inflammatory M1 after exposure to LPS (**Figure 2A**). IL-38 blocking significantly aggravated the inflammation and increased the differentiation from M2 to M1 macrophages (11). Interestingly, another study reported that dendritic cells (DCs) regulate the expression of inflammatory factors by affecting the infiltration of mononuclear macrophages in the infarcted heart during ventricular remodeling after MI (47).

NOD-like receptor thermal protein domain-associated protein 3 (NLRP3) inflammasome, an effective pro-inflammatory mediator of the innate immune system, is also a regulator of various inflammatory diseases such as MI, atherosclerosis, and diabetes (48–51). NLRP3 inflammasome consists of NLRP3 protein, ASC, and caspase 1. Once activated, cytokine precursors (i.e., promoting IL-1β) are converted to a biologically active form and trigger inflammatory responses (52). The NLRP3 inflammasome protein in macrophages cotreated with IL-38 and LPS was downregulated and returned to levels similar to those of control cells that were not treated with LPS (11). IL-38 can significantly inhibit the activation of NLRP3, thus showing a potent anti-inflammatory activity (11) (**Figure 2B**).

However, recent studies have shown that IL-38 can also exhibit anti-inflammatory effects by binding to interleukin 1 receptor accessory protein-like 1 (IL-1RAPL1) on the surface of LPS-activated macrophages (28, 53). Interestingly, the intact recombinant IL-38 binds to IL-1RAPL1, activates the downstream JNK/AP1 pathway, and increases the production of IL-6 to act as a stimulator (**Figure 2C**). Conversely, truncated IL-38 reduces JNK/AP1 signaling and restricts Th17 activation by lowering IL-6 and IL-8 levels (53–55). Although most *in vitro* experiments have reported that IL-38 can regulate the function of macrophages and play an anti-inflammatory role, the specific binding sites of IL-38 on macrophages are not completely known. These results also indicated that IL-38 binding receptors are diverse; hence, how IL-38 acts on macrophages in the myocardium needs to be further studied and discussed.

## Regulation of Interleukin-38 in Macrophages Has Therapeutic Potential in Myocardial Ischemia–Reperfusion Injury

Reperfusion therapy for ischemia caused by MI, such as percutaneous coronary intervention and intravenous thrombolytic therapy, can improve myocardial ischemia, limit the MI size in a timely and effective manner, and quickly restore the blood supply to the heart. However, in the process of blood flow restoration of the ischemic myocardium, reperfusion injury may occur because of cellular swelling and myofibrillary contracture, thus resulting in poor prognoses such as local acute inflammatory reaction, and metabolic disorder, apoptosis or necrosis, and even cardiac insufficiency (7, 56, 57). As the main cell of an inflammatory response, macrophages play an important role in this process (58). As mentioned earlier, M1 macrophages, an inflammatory cell, damage cardiac tissues and cells by releasing inflammatory mediators and proteases in the early stage of reperfusion (59). A study has reported that in the myocardium, myocardial ischemia–reperfusion injury is mainly caused by macrophages polarized by Dectin-1 (60). Interestingly, soluble receptors for advanced glycation end products can improve cardiac function after ischemia–reperfusion in mice *via* IFN-γ production and promote macrophage infiltration and differentiation into the M1 type (61). These results suggested that M1 macrophages not only participate in inflammatory reactions and cause cardiac damage during ischemia–reperfusion but also have a protective effect on the heart during ischemia. On the contrary, M2 macrophages exhibit anti-inflammatory and tissue repair properties by producing high levels of anti-inflammatory cytokines and fibro-progenitors and alleviate myocardial ischemia–reperfusion injury. Our previous study showed that M2 macrophages alternatively activated by Chemerin15 can protect against myocardial ischemia–reperfusion injury in mice by significantly controlling pro-inflammatory cytokines and observably increasing the level of the anti-inflammatory cytokine IL-10 (62). Evidence has indicated that M2 macrophages have a protective effect on the myocardium after ischemia–reperfusion; hence, effectively restricting M1 and promoting differentiation of M2 macrophages may be a therapeutic target for improving myocardial ischemia–reperfusion injury in the future. The aforementioned promotion of IL-38 on M1-to-M2 transformation reflects the potential therapeutic effect of IL-38 on myocardial ischemia–reperfusion injury.

## Interleukin-38 Ameliorates Malignant Ventricular Remodeling After Myocardial Infarction

**Figure 3:**
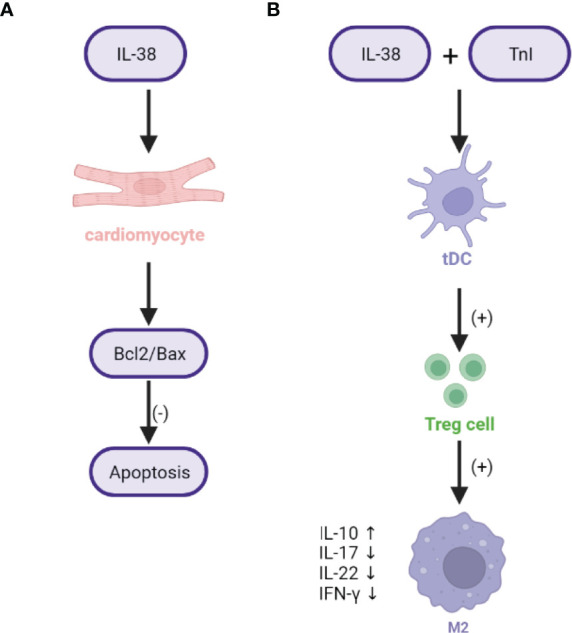
The mechanisms of IL-38 and ischemic injury and repair after MI. **(A)** The mechanism of IL-38 on cardiomyocytes during ventricular remodeling. **(B)** The mechanism of IL-38 on tDCs during ventricular remodeling. MIRI, myocardial ischemia–reperfusion injury; IL-38, interleukin-38; IFN-γ, interferon-γ; tDCs, tolerogenic dendritic cells; Treg cells, regulatory T cells; (+), promoting effect; (-), inhibition.

Although an early coronary reperfusion strategy can significantly improve survival in patients with MI, reducing the risk of heart failure after MI is essential. The emerging understanding of the role of macrophages in inflammation after MI suggests that these cells are necessary for wound healing and the production of stable scars for ventricular remodeling; however, excess pro-inflammatory macrophages are also harmful (8, 34, 45, 63). Therefore, timely suppression of excessive inflammation after MI is necessary to prevent malignant ventricular remodeling and ensure the optimal formation of a supportive scar in the infarct area. In our previous study, we reported that IL-38 mRNA expression was significantly increased in the boundary region of the infarcted heart in mice and reached a peak at 24 h. Double immunofluorescence experiments on major cells involved in the development of MI showed that IL-38 was mainly expressed in cardiomyocytes and also detected in CD68 macrophages 7 days after MI. Thus, both cardiomyocytes and macrophages can be the sources of IL-38 after MI (10). Combined with the previously observed increase in circulating IL-38 levels in ST-segment elevation MI patients, IL-38 may be implicated in the development of MI (9). The infiltration of CD45^+^ cells in the infarcted heart of mice treated with recombinant IL-38 on the seventh day was significantly reduced compared with that in the control group. Especially, the macrophages of CD45^+^CD11b^+^F4/80^+^ and CD45^+^CD11b^+^F4/80^+^CD86^+^ were reduced after IL-38 treatment, whereas the macrophages of CD45^+^CD11b^+^F4/80^+^CD86^-^ were not increased. At the same time, immunohistochemical staining results of mouse hearts showed that CD68^+^ macrophages in the IL-38 treatment group were significantly reduced compared with those in the control group. These results showed that IL-38 can ameliorate ventricular remodeling after MI by cutting down M1 macrophages to decrease the secretion of pro-inflammatory molecules, rather than increasing the number of M2 macrophages (10, 64). Furthermore, high IL-38 levels in ST-segment elevation MI patients may be an adaptive mechanism to prevent the progression of harmful ventricular remodeling after MI. B-cell lymphoma-2 (Bcl-2) family proteins are upstream regulators of mitochondrial cytochrome C release, and the ratio of Bcl-2-associated X (Bax) to Bcl-2 is an important determiner of cell apoptosis susceptibility (65). IL-38 overexpression in cultured cardiomyocytes reduced the mRNA level of the pro-apoptotic molecule Bax and increased the expression of the anti-apoptotic molecule Bcl-2 (10), suggesting that IL-38 may affect cardiomyocyte apoptosis by regulating the Bcl-2/Bax pathway (**Figure 3A**).

In addition to acting on cardiomyocytes and macrophages, IL-38 exhibits anti-inflammatory effects by regulating the function of DCs. Anzai et al. (47) reported that the infiltration of M2 macrophages of F4/80^+^CD206^+^ was reduced in the DC-deficient MI model, whereas the infiltration of M1 macrophages of F4/80^+^CD206^-^ was increased. Choo et al. (66) observed the expression of the cell markers of M1 and M2 macrophages in the MI model under tolerogenic dendritic cell (tDC) treatment. They found that the expression of M1 markers (IL-1β, IL-6, IL-12b, and TNF-α mRNA) decreased significantly by day 3. On the contrary, the expression of M2 markers (arginase-1, Mrcl1, Mgl1, and Mgl2) increased by day 5 (66). Moreover, regulatory T (Treg) cells may inhibit inflammation by controlling the secretion of anti-inflammatory factors such as IL-10 and TGF-β (67). Choo et al. (66) reported that tDCs induce the production of myocardium-directed Treg cells, which then orchestrate healing by affecting the polarization of macrophages to initiate repair programs and augmenting new angiogenesis in the infarcted heart. The abundance of Treg cells in mice with MI steered the macrophage differentiation toward the M2 type, thus resulting in favorable ventricular remodeling (**Figure 3B**). Excessive effective T cells and insufficient recruitment of Treg cells can lead to malignant ventricular remodeling after MI (68). The ability of IL-37-induced DCs to activate naive T cells and induce Treg cells has been reported (69), whereas tolerant DCs induced by IL-38 and TnI show stronger tolerance (10). We found that IL-38 inhibited LPS-induced maturation of DCs, thus resulting in lower expression of cell surface molecules and inflammatory factors than that in LPS-induced DCs that were not treated with IL-38 (10). These results suggested that IL-38 may be involved in ventricular remodeling after MI *via* the regulation of DCs to Treg cells and macrophage polarization. Therefore, IL-38 has the potential as a protective factor in ameliorating malignant ventricular remodeling after MI.

## Conclusions

Although IL-38 has not been known for a long time, it is actively studied in various inflammatory diseases. In the context of MI, IL-38 may regulate the release of inflammatory factors by regulating the macrophage phenotype (10, 11) and the function of DCs (10, 69) and play a similar function to IL-36Ra by antagonizing the relevant receptors in the IL-36 pathway (16, 70). Thus, it regulates cardiac function and ventricular remodeling after MI, even myocardial injury after ischemia–reperfusion therapy. Most studies have reported that regulating the phenotypic transformation of M1 and M2 macrophages can promote the enhancement of cardiac angiogenesis, improve cardiac function, and reduce the infarct area after MI, thus facilitating a better prognosis (71–73). Therefore, the regulation of macrophages by IL-38 indicates its potential therapeutic value and requires more research. Although several studies have reported the role of IL-38 in autoimmune diseases and inflammation-related diseases, IL-38-related signaling pathways and anti-inflammatory mechanisms are unclear. A series of issues such as the production source of IL-38 in different environments and the main functional cells, the possible different inflammatory effects of different concentrations of IL-38, and whether IL-38 directly acts on macrophages under myocardial ischemia need to be further studied.

## Author Contributions

ZL, YD, and YP wrote the article. KY and QZ edited the article. YC, RZ, and JY finished the figures. QD, CP, and YZ provided feedback and guidance. All authors contributed to the article and approved the submitted version.

## Funding

This work was supported by the National Natural Science Foundation of China (grant numbers 82070310, 81770273, 81900400, 81900270, 82100339).

## Conflict of Interest

The authors declare that the research was conducted in the absence of any commercial or financial relationships that could be construed as a potential conflict of interest.

## Publisher’s Note

All claims expressed in this article are solely those of the authors and do not necessarily represent those of their affiliated organizations, or those of the publisher, the editors and the reviewers. Any product that may be evaluated in this article, or claim that may be made by its manufacturer, is not guaranteed or endorsed by the publisher.
